# Supplementary material for: Spatio-molecular domains identified in the mouse subthalamic nucleus and neighboring glutamatergic and GABAergic brain structures
Source: Commun Biol. 2020 Jul 3;3:338. doi: 10.1038/s42003-020-1028-8 (PMC7334224; doi:10.1038/s42003-020-1028-8)
Supplement: Supplementary file 1 — Supplementary Information [file 42003_2020_1028_MOESM1_ESM.pdf]

**Supplementary Information**

# Spatio-molecular domains identified in the mouse subthalamic nucleus and neighboring glutamatergic and GABAergic brain structures

Supplementary figures: 6

Supplementary tables: 3

Supplementary Figure 1 related to Figure 1

**a**

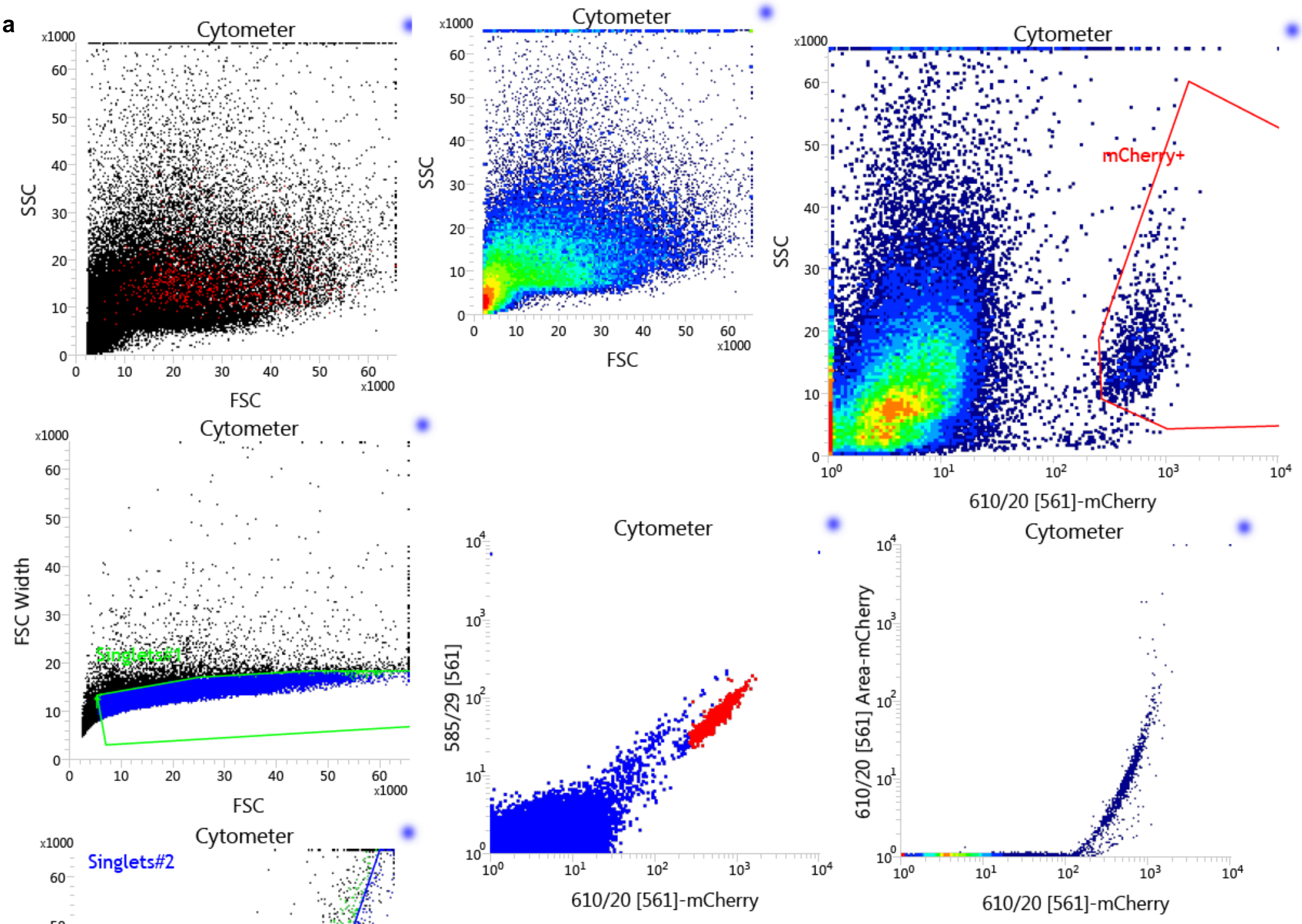


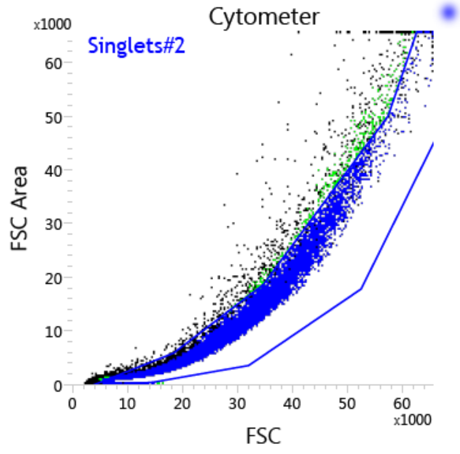


**b**

Statistics: Cytometer

| Populations | Events | % Total | % Parent | FSC Median | SSC Median | 610/20 [5... Median |
|---|---|---|---|---|---|---|
| All Events | 50,000 | 100.00% | #### | 9,089 | 9,067 | 4 |
| Singlets#1 | 27,895 | 55.79% | 55.79% | 17,344 | 11,796 | 5 |
| Singlets#2 | 27,244 | 54.49% | 97.67% | 17,275 | 11,682 | 5 |
| mCherry+ | 731 | 1.46% | 2.68% | 27,262 | 15,460 | 538 |

**Supplementary Figure 1 related to Figure 1: Information related to the sorting layout and sorting parameters. (a)** FACS methodology and gating parameters. **(b)** Statistics cytometer showing percentage of sorted population.

Supplementary Figure 2 related to Figure 1

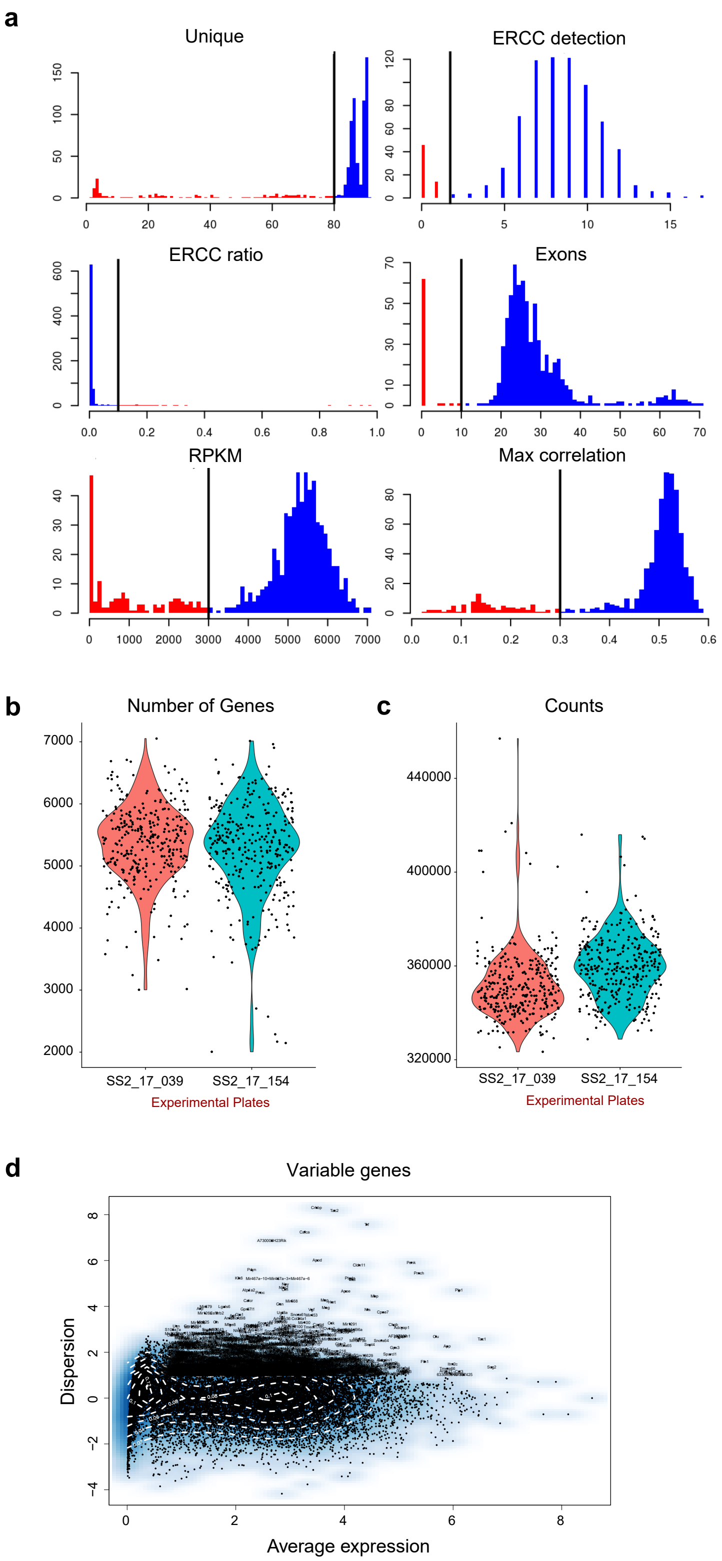


**Supplementary Figure 2 related to Figure 1: Quality control of snRNASeq experiment. (a)** Quality control of the two experimental plates with histograms of percent uniquely mapping (Unique), Spike-in detection (ERCC), Spike-in ratio (ERCC-ratio), percent mapped to exons (Exons), detected genes with RPKM>1 (RPKM) and highest correlation to another cell (Max correlation). Nuclei which failed cutoffs are colored in red. **(b)** Violin plots showing the number of detected genes of each nuclei (5384) across the two experiment plates according to Seurat unsupervised clustering. **(c)** Violin plots showing the average number of transcripts (354578) across the two experiment plates. **(d)** Identification of highly variable genes (out of 18697 genes 1776 selected as variable). The dispersion of the genes (variance/log-average expression) is plotted against the average expression (normalized log-expression, on x-axis). The minimum threshold to be considered highly variable was set at a dispersion of 1 on the y-axis.

Supplementary Figure 3 related to Figure 2

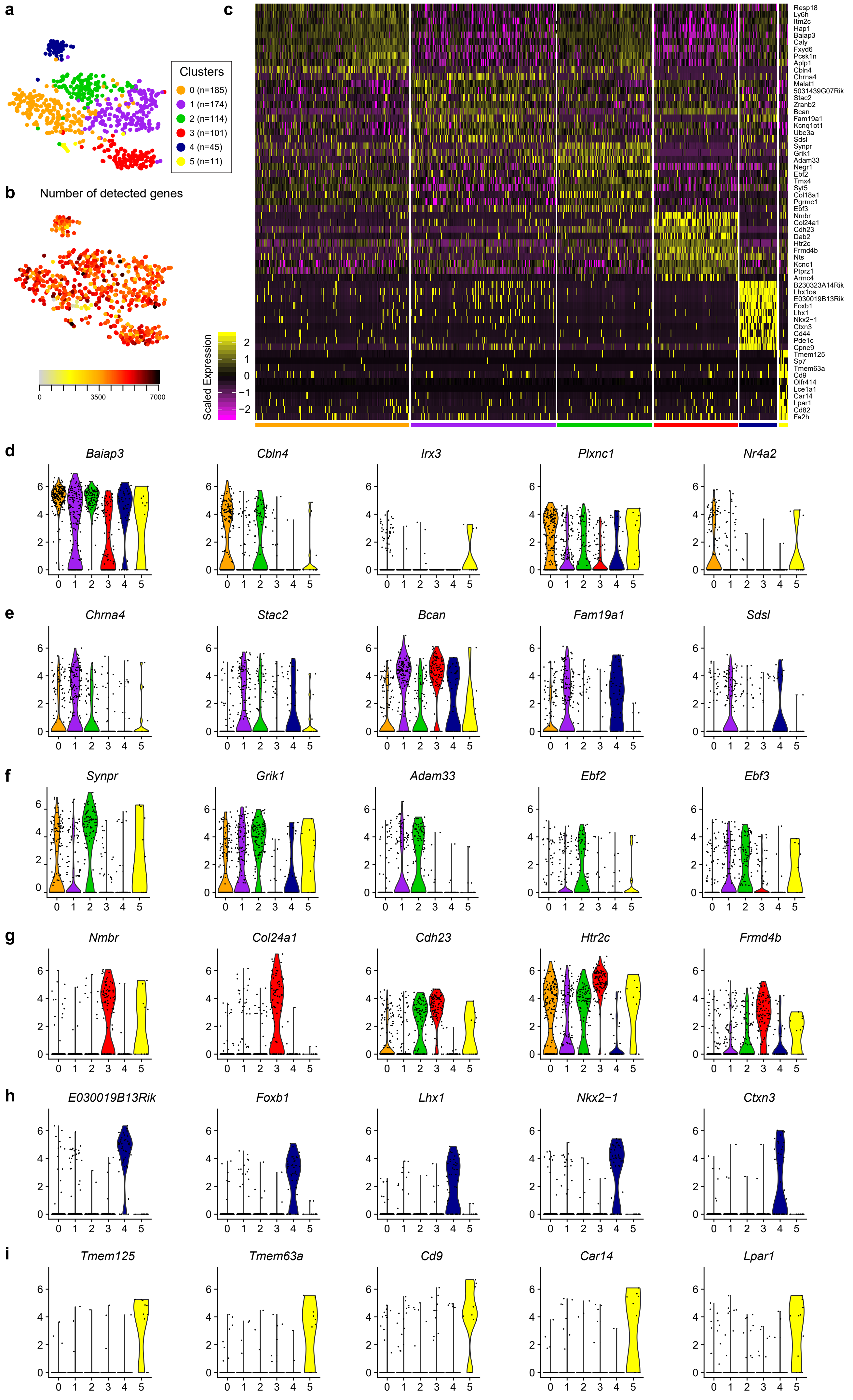

**Supplementary Figure 3 related to Figure 2: Identification of 6 different glutamatergic *Pitx2*-positive subpopulations.** **(a)** t-SNE plot with 6 clusters (orange, purple, green, red, blue and yellow). **(b)** Number of detected genes per nuclei (expression levels ranging from light grey (low) to black (high). **(c)** Heatmap showing the top ten genes considered unique for and characterizing each cluster **(d-i)** Violin plots of 5 of the top 10 identified discriminatory markers of each cluster.

Supplementary Figure 4 related to Figure 2

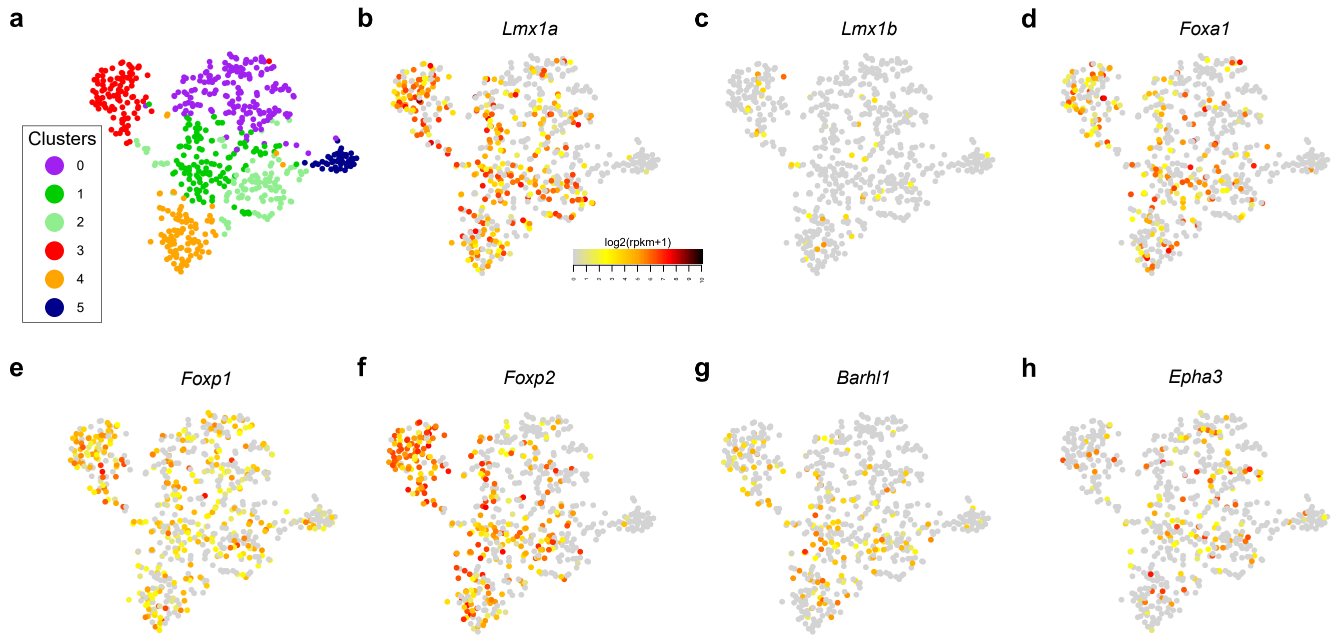


**Supplementary Figure 4 related to Figure 2: t-SNE of previously established markers for the developing STN. (a)** t-SNE plot with 6 clusters (purple cluster0; green cluster1; light-green cluster2; red cluster3; orange cluster4 and blue cluster5). **(b-h)** Expression levels of *Lmx1a*, *Lmx1b*, *Foxa1*, *Foxp1*, *Foxp2*, *Barhl1* and *Epha3*, genes previosuly associated with the STN (References 1-3), visualized in the t-SNE plot.

Supplementary Figure 5 related to Figure 2

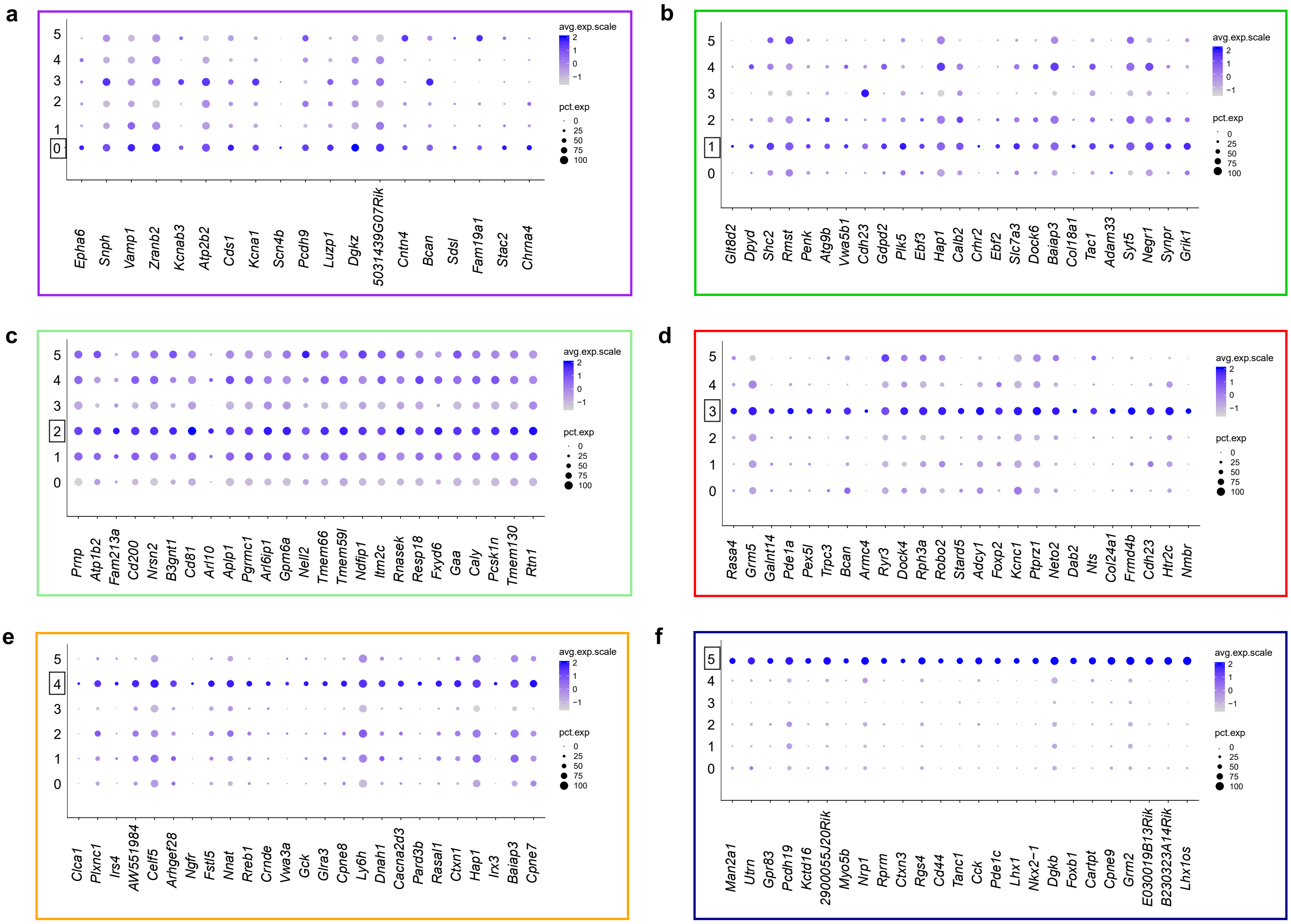


**Supplementary Figure 5 related to Figure 2: Dotplot of upregulated genes across each cluster (a-f)** Dotplots of up to 25 significantly upregulated genes of each cluster *vs* the rest using MAST R package.

Supplementary Figure 6 related to Figure 2

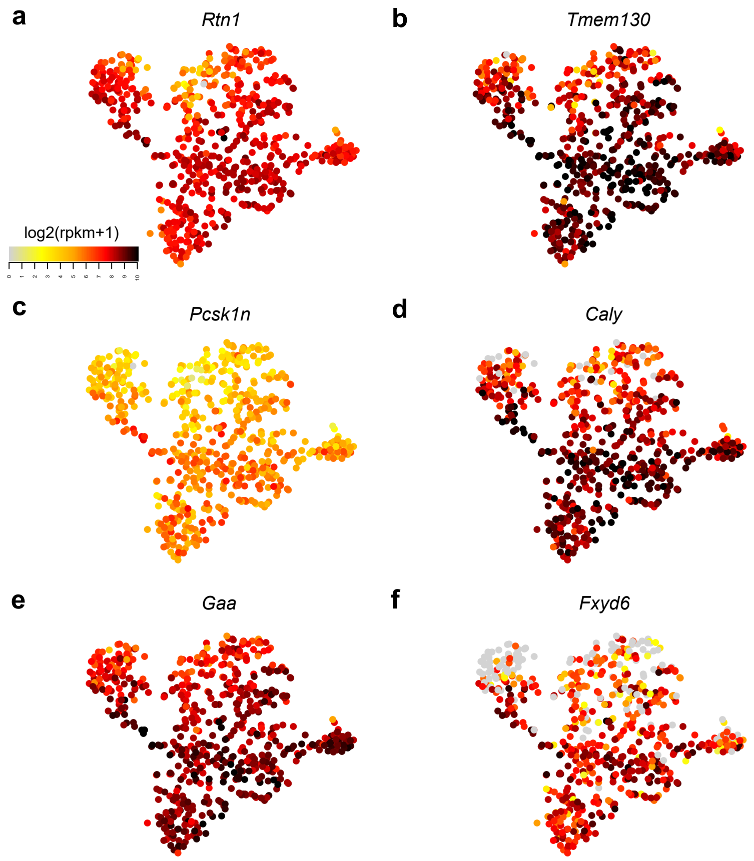


**Supplementary Figure 6 related to Figure 2: t-SNE-plots of selected genes showing gradient of expression.** **(a)** *Rtn1* **(b)** *Tmem130* **(c)** *Pcsk1n* **(d)** *Caly* **(e)** *Gaa* **(f)** *Fxyd6* showing gradient of expression along the t-SNE-2 axis.

**Supplementary Table 1: PCR primer sequences used for genotyping of transgenic mice employed in the study**.

| Transgene | Direction | PCR primer sequence |
|---|---|---|
| Pitx2-Cre | Fw | 5'-ACGAGTGATGAGGTTCGCAAGA-3' |
| Pitx2-Cre | Rev | 5'-ACCGACGATGAAGCATGTTTAG-3' |
| R26-Rpl10a | Fw (mutant) | 5'- TAC ACC ATC GTG GAA CAG TAC -3' |
| R26-Rpl10a | Rev (mutant) | 5'- GTA GTT CTT CAG GCT GAT CTG -3' |
| R26-wt | Fw (WT) | 5'- GCG GAT CAC AAG CAA TAA TA -3' |
| R26-wt | Rev (WT) | 5'- TTT CTG GGA GTT CTC TGC TG -3' |

**Supplementary Table 2: Table of NM accession number and primer sequences for riboprobe synthesis of mRNAs selected for analysis in mouse brain tissue by fluorescent *in situ* hybridization (FISH)**

| PCR Primer Name | PCR Primer Sequence | NM | bases |
|---|---|---|---|
| Pitx2-S-T3 | AATTAACCCTCACTAAAGGGACCCCGCCCAACTCCATCTCA | NM_001042504.2 | 792-1579 |
| Pitx2-AS-T7 | TAATACGACTCACTATAGGGTCCTTTGCTCGCAAGCGAAAAATC | | |
| Nmbr-S-T3 | AATTAACCCTCACTAAAGGGACCCCAGGTCTCTCTCCAACC | NM_008703.3 | 348-1280 |
| Nmbr-AS-T7 | TAATACGACTCACTATAGGGCTCAGAACCCGGGCCACTA | | |
| Col24a1-S-T3 | AATTAACCCTCACTAAAGGGAAGGGACCTCCTGGCACTGAG | NM_027770.3 | 3722-4593 |
| Col24a1-AS-T7 | TAATACGACTCACTATAGGGGCCGTCGACTCCAGGGTATC | | |
| Kcnab3-S-T3 | AATTAACCCTCACTAAAGGGAGCAGCTGCCTGAGCTCTACC | NM_010599.4 | 1228-2184 |
| Kcnab3-AS-T7 | TAATACGACTCACTATAGGGCAGGCAGGAGGCAGGAGTC | | |
| Stxbp2-S-T3 | AATTAACCCTCACTAAAGGGAATGCCAACGTGCAGTCGTACA | NM_001357168.1 | 1342-1907 |
| Stxbp2-AS-T7 | TAATACGACTCACTATAGGGGGGAAGACAGAAGGGAAGCGTTT | | |
| Baiap3-S-T3 | AATTAACCCTCACTAAAGGGAGACCACGACTGGCTGTCCAC | NM_001163270.1 | 3415-4384 |
| Baiap3-AS-T7 | TAATACGACTCACTATAGGGGAGCCCTTCAGCCTGGTCTC | | |
| Cacna2d3-S-T3 | AATTAACCCTCACTAAAGGGATGGTGAGGTGGAAGGAGCTG | NM_009785.1 | 2731-2750 |
| Cacna2d3-AS-T7 | TAATACGACTCACTATAGGGGGCCTGAAGACTCGATGCAC | | |
| Synpr-S-T3 | AATTAACCCTCACTAAAGGGACTAGATGACTATTTGATGAATCACATCATGCATGC | NM_028052.4 | 1453-2458 |
| Synpr-AS-T7 | TAATACGACTCACTATAGGGATAACTTTATTGAGGTCAAATAGATATCACAGTACAGG | | |
| Nxph4-S-T3 | AATTAACCCTCACTAAAGGGACTCCCGGAATGGCTCCTCT | NM_183297.2 | 137-991 |
| Nxph4-AS-T7 | TAATACGACTCACTATAGGGCTGCACCAGTTTGTAGTCAAAACTGAGG | | |
| Fgf11-S-T3 | AATTAACCCTCACTAAAGGGACTAATCAGTACTGAACGGATCAAGAACCACT | NM_010198.3 | 2205-3005 |
| Fgf11-AS-T7 | TAATACGACTCACTATAGGGAGCCCCTCCCTTAGGACCTC | | |
| Glra3-S-T3 | AATTAACCCTCACTAAAGGGAGGAGATTCTGGCTCTTGGACCTAGT | NM_080438.3 | 43-692 |
| Glra3-AS-T7 | TAATACGACTCACTATAGGGGAGGACCTTTGAAGTTGGGTCTGATTCT | | |
| Adcyap1-S-T3 | AATTAACCCTCACTAAAGGGAGCTTCCCTGGGATCAGACC | NM_009625.3 | 547-1510 |
| Adcyap1-AS-T7 | TAATACGACTCACTATAGGGAGCCTGCCCCAGACTCAGA | | |
| Nxph1-S-T3 | AATTAACCCTCACTAAAGGGAACTACAACTATCACAGTGACACACCTTACTTTC | NM_008751.5 | 1008-1783 |
| Nxph1-AS-T7 | TAATACGACTCACTATAGGGAAGATAAGACAGACACTTATAACTTACATGGTGTCTGC | | |

| | | | |
|---|---|---|---|
| Htr2c-S-T3 | AATTAACCCTCACTAAAGGGAGCAGGTGACCAGAATGAGGCACA | NM_008312.4 | 2456-3446 |
| Htr2c-AS-T7 | TAATACGACTCACTATAGGGGAGAGCAGCAGGCCCACGAA | | |
| Calb2-S-T3 | AATTAACCCTCACTAAAGGGACGCAGCAGCAGCCCCCTTAC | NM_007586.1 | 80-793 |
| Calb2-AS-T7 | TAATACGACTCACTATAGGGTGGTGAGCTGTTGGATGTTCATCTCC | | |
| Vglut2-S-T3 | AATTAACCCTCACTAAAGGGACCTTGGGCAGACCCTGAGGAA | NM_080853.3 | 2315-3244 |
| Vglut2-AS-T7 | TAATACGACTCACTATAGGGGGGGGAGCATGGAGCATACCC | | |
| Gad1-S-T3 | AATTAACCCTCACTAAAGGGACACGCCTTCGCCTGCAACCT | NM_008077.5 | 284-819 |
| Gad1-AS-T7 | TAATACGACTCACTATAGGGGGTGACCTGTGCGAACCCCG | | |

## Supplementary Table 3: Full names of mRNAs analyzed in mouse brain sections by fluorescent *in situ* hybridization (FISH)

| **Abbreviation, mRNA** | **Full name** |
|---|---|
| Adcyap1 | Adenylate Cyclase Activating Polypeptide 1 |
| Baiap3 | BAI1 Associated Protein 3 |
| Cacna2d3 | Calcium Voltage-Gated Channel Auxiliary Subunit Alpha2delta 3 |
| Calb2 | Calbindin 2 |
| Col24a1 | Collagen Type XXIV Alpha 1 Chain |
| Fgf11 | Fibroblast Growth Factor 11 |
| Gad1 | Glutamate decarboxylase 1 |
| Glra3 | Glycine Receptor Alpha 3 |
| Htr2c | 5-Hydroxytryptamine Receptor 2C |
| Kcnab3 | Potassium Voltage-Gated Channel Subfamily A Regulatory Beta Subunit 3 |
| Nmbr | Neuromedin B Receptor |
| Nxph1 | Neurexophilin 1 |
| Nxph4 | Neurexophilin 4 |
| Pitx2 | Paired Like Homeodomain 2 |
| Pvalb2 | Parvalbumin |
| Stxbp2 | Syntaxin Binding Protein 2 |
| Synpr | Synaptoporin |
| Tac1 | Tachykinin Precursor 1 |
| Vglut2 | Vesicular glutamate transporter 2 |

## Supplementary References


1. Skidmore, J. M., Cramer, J. D., Martin, J. F. & Martin, D. M. Cre fate mapping reveals lineage specific defects in neuronal migration with loss of Pitx2 function in the developing mouse hypothalamus and subthalamic nucleus. Mol. Cell. Neurosci. 37, 696–707 (2008).

2. Gasser, E., Johannssen, H. C., Rülicke, T., Zeilhofer, H. U. & Stoffel, M. Foxa1 is essential for development and functional integrity of the subthalamic nucleus. Sci Rep 6, 38611 (2016).

3. Kee, N. et al. Single-Cell Analysis Reveals a Close Relationship between Differentiating Dopamine and Subthalamic Nucleus Neuronal Lineages. Cell Stem Cell 20, 29–40 (2017).
