# Supplementary material for: Spatio-molecular domains identified in the mouse subthalamic nucleus and neighboring glutamatergic and GABAergic brain structures
Source: Commun Biol. 2020 Jul 3;3:338. doi: 10.1038/s42003-020-1028-8 (PMC7334224; doi:10.1038/s42003-020-1028-8)
Supplement: Supplementary file 2 — Description of Additional Supplementary Files [file 42003_2020_1028_MOESM2_ESM.pdf]

Supplementary Data 1: Differential expression analysis. Table (excel format) showing data obtained by the Model-based Analysis of Single-cell Transcriptomics (MAST) method. Each cluster was compared versus the rest using a p adjusted value of <0.01.

Supplementary Data 2: Differential expression analysis. Table (excel format) showing data obtained by the Model-based Analysis of Single-cell Transcriptomics (MAST) method comparing specific clusters.

Supplementary Data 3: Source data underlying Figures 1 and 2.
