# Supplementary material for: Spatio-molecular domains identified in the mouse subthalamic nucleus and neighboring glutamatergic and GABAergic brain structures
Source: Commun Biol. 2020 Jul 3;3:338. doi: 10.1038/s42003-020-1028-8 (PMC7334224; doi:10.1038/s42003-020-1028-8)
Supplement: Supplementary file 6 — Reporting Summary [file 42003_2020_1028_MOESM6_ESM.pdf]

nature research

Corresponding author(s): Åsa Wallén-Mackenzie & Maria Papathanou

Last updated by author(s): Aug 29, 2019

Reporting Summary

Nature Research wishes to improve the reproducibility of the work that we publish. This form provides structure for consistency and transparency in reporting. For further information on Nature Research policies, see Authors & Referees and the Editorial Policy Checklist.

Statistics

For all statistical analyses, confirm that the following items are present in the figure legend, table legend, main text, or Methods section.

n/a	Confirmed	
☐	☒	The exact sample size (*n*) for each experimental group/condition, given as a discrete number and unit of measurement
☒	☐	A statement on whether measurements were taken from distinct samples or whether the same sample was measured repeatedly
☒	☐	The statistical test(s) used AND whether they are one- or two-sided<br>*Only common tests should be described solely by name; describe more complex techniques in the Methods section.*
☒	☐	A description of all covariates tested
☒	☐	A description of any assumptions or corrections, such as tests of normality and adjustment for multiple comparisons
☒	☐	A full description of the statistical parameters including central tendency (e.g. means) or other basic estimates (e.g. regression coefficient) AND variation (e.g. standard deviation) or associated estimates of uncertainty (e.g. confidence intervals)
☒	☐	For null hypothesis testing, the test statistic (e.g. *F*, *t*, *r*) with confidence intervals, effect sizes, degrees of freedom and *P* value noted<br>*Give P values as exact values whenever suitable.*
☒	☐	For Bayesian analysis, information on the choice of priors and Markov chain Monte Carlo settings
☒	☐	For hierarchical and complex designs, identification of the appropriate level for tests and full reporting of outcomes
☒	☐	Estimates of effect sizes (e.g. Cohen's *d*, Pearson's *r*), indicating how they were calculated

Our web collection on statistics for biologists contains articles on many of the points above.

Software and code

Policy information about availability of computer code

Data collection	Single-nuclei RNA-sequencing data was collected using Illumina HiSeq 2000.<br>In situ hybridization images were captured using the NanoZoomer 20.2-HT-0 with a 20x objective and the Ndp2-view software (Hamamatsu)
Data analysis	RefSeq and Ensemble gene annotations were computed as described by Rämskold et al 2009 and aligned using MULTo and STAR aligner v2.3.0. Sequencing data were analyzed using the Seurat package 2.34 (based on Wilcoxon rank sum test and Bonferroni correction test) and MAST 1.8.2 for differential expression (exact p value and adjusted p values for each cluster; p adjusted value <0.01 criteria for up and down regulated genes). Data were visualized using Violin Plots, Dotplots and t-SNE plots. Brain slides processed for in situ hybridization were digitally captured using Hamamatsu scanner and analyzed using the ndp2 software. Semi-quantitative analysis was made by manual counting in 2-3 animals, several sections per probe combination per animal for each anatomically defined area.

For manuscripts utilizing custom algorithms or software that are central to the research but not yet described in published literature, software must be made available to editors/reviewers. We strongly encourage code deposition in a community repository (e.g. GitHub). See the Nature Research guidelines for submitting code & software for further information.

Data

Policy information about availability of data

All manuscripts must include a data availability statement. This statement should provide the following information, where applicable:

- Accession codes, unique identifiers, or web links for publicly available datasets
- A list of figures that have associated raw data
- A description of any restrictions on data availability

Raw data related to Figures 1-2 and supplementary figures 1-6 have been uploaded to GEO with accession number GSE133953. The data is restricted but reviewer access tokens are available #snsgogmzridbab. All in situ hybiridization data is presented as figures and tables in the manuscript.

Field-specific reporting

Please select the one below that is the best fit for your research. If you are not sure, read the appropriate sections before making your selection.

☒ Life sciences ☐ Behavioural & social sciences ☐ Ecological, evolutionary & environmental sciences

For a reference copy of the document with all sections, see nature.com/documents/nr-reporting-summary-flat.pdf

Life sciences study design

All studies must disclose on these points even when the disclosure is negative.

Sample size	4 animals (Pitx2-Cre/mCherry-positive) were used for collection of nuclei for the analysis and 2 animals (Pitx2-Cre/mCherry-negative) were used for setting the cell sorter, serving as negative control. Nuclei from each animal were individually extracted and sorted and were never pooled. No statistical methods were used to predetermine sample size.
Data exclusions	Only exons were included in the analysis. Parameters for quality control consisted of uniquely mapping reads (<80%), ERCC detection (<mean -2 standard deviations), ERCC-ratio (>0.1), fraction of reads mapping to exons (<10%), detected with RPKM >1 (>3000 and < mean + 2 standard deviations) and a maximum correlation to another nuclei (< 0.3). Nuclei that failed in two of these QC parameters were excluded from further analysis. For the Seurat object a minimum of 3 nuclei and a minimum of 200 genes was the preliminary criterion. Nuclei expressing a high proportion of genes representing the oligodendrocyte lineage were excluded.<br>Fluorescent in situ hybridization was performed on multiple sections derived from 2-3 mice per probe combination. Semi-quantitative analysis was made by manual counting in 2-3 animals, several sections per probe combination per animal for each anatomically defined area. No data was excluded from the analysis.
Replication	Each experimental 384 well plate comprised of two separate animals with 191 cells from each animal. Plate 1 contained single sorted nuclei from animal 1 and 2 and plate 2 contained nuclei from animals 3 and 4. During nuclear extraction and sorting, the animals were kept as independent samples and were never pooled, giving rise to 4 biological replicates. Both plates and nuclei from all 4 animals were represented in the identified clusters. Each nuclei represents one data set.<br>Fluorescent in situ hybridization: All expression patterns were verified by analysis in multiple sections from 2-3 mice per probe combination.
Randomization	Randomization of samples was not applicable in this study.
Blinding	Blinding of samples was not applicable in this study.

Reporting for specific materials, systems and methods

We require information from authors about some types of materials, experimental systems and methods used in many studies. Here, indicate whether each material, system or method listed is relevant to your study. If you are not sure if a list item applies to your research, read the appropriate section before selecting a response.

Materials & experimental systems

n/a	Involved in the study	
☒	☐	Antibodies
☒	☐	Eukaryotic cell lines
☒	☐	Palaeontology
☐	☒	Animals and other organisms
☒	☐	Human research participants
☒	☐	Clinical data

Methods

n/a	Involved in the study	
☒	☐	ChIP-seq
☐	☒	Flow cytometry
☒	☐	MRI-based neuroimaging

Animals and other organisms

Policy information about studies involving animals; ARRIVE guidelines recommended for reporting animal research

Laboratory animals	Transgenic male mice of postnatal day 28 were used for the study generated by crossing Pitx2-Cre (Skidmore et al 2014) and the reporter Gt(ROSA)-26Sor-mCherry-Rpl110a (Hupe et al 2014). Wildtype mice (c57bl/6n, Taconic, Dk) at same postnatal stage were used for in situ hybridization analysis.
Wild animals	No wild animals were used.
Field-collected samples	No samples collected from the field.
Ethics oversight	All experimental procedures followed the guidelines and recommendations of the European and Swedish animal protection and legislation and. were approved by the local animal ethical committee (Region Uppsala).

Note that full information on the approval of the study protocol must also be provided in the manuscript.

Flow Cytometry

Plots

Confirm that:

☒ The axis labels state the marker and fluorochrome used (e.g. CD4-FITC).

☒ The axis scales are clearly visible. Include numbers along axes only for bottom left plot of group (a 'group' is an analysis of identical markers).

☒ All plots are contour plots with outliers or pseudocolor plots.

☒ A numerical value for number of cells or percentage (with statistics) is provided.

Methodology

Sample preparation	Detailed description of sample preparation is found in Material and Methods under "Nuclear extraction and FACS sorting of mCherry-tagged nuclei".
Instrument	Nuclei were sorted using BD Influx System.
Software	FACs samples were analyzed using the BD FACS Software.
Cell population abundance	The abundance and fraction of mCherry-positive population can be found in Supplementary figure 1.
Gating strategy	Forward and side-scatter gating was applied using a 633nm later with a 610/20 [561] filter. See supplementary figure 1.

☒ Tick this box to confirm that a figure exemplifying the gating strategy is provided in the Supplementary Information.
